# Nardosinone Improves the Proliferation, Migration and Selective Differentiation of Mouse Embryonic Neural Stem Cells

**DOI:** 10.1371/journal.pone.0091260

**Published:** 2014-03-10

**Authors:** Ze-Hui Li, Wei Li, Jin-Li Shi, Min-Ke Tang

**Affiliations:** 1 Department of Biomedical Science, College of Medicine, University of Toledo, Toledo, Ohio, United States of America; 2 Department of Pharmacology, Beijing University of Chinese Medicine, Beijing, China; Baylor College of Medicine, United States of America

## Abstract

In this study, we investigated the impact of Nardosinone, a bioactive component in Nardostachys root, on the proliferation and differentiation of neural stem cells. The neural stem cells were isolated from cerebrums of embryonic day 14 CD1 mice. The proliferation of cells was monitored using the cell counting kit-8 assay, bromodeoxyuridine incorporation and cell cycle analysis. Cell migration and differentiation were investigated with the neurosphere assay and cell specific markers, respectively. The results showed that Nardosinone promotes cells proliferation and increases cells migration distance in a dose-dependent manner. Nardosinone also induces the selective differentiation of neural stem cells to neurons and oligodendrocytes, as indicated by the expression of microtubule-associated protein-2 and myelin basic protein, respectively. Nardosinone also increases the expression of phospho-extracellular signal-regulated kinase and phospho-cAMP response element binding protein during proliferation and differentiation. In conclusion, this study reveals the regulatory effects of Nardosinone on neural stem cells, which may have significant implications for the treatment of brain injury and neurodegenerative diseases.

## Introduction

Nardostachys root was first recorded as a Chinese medicine in the book *A Supplement to Materia Medica* in 741 A.D. Since then, this herbal medicine has been widely used in the clinical practice of Chinese medicine for the treatment of a variety of illnesses. Pharmacological studies suggest that extracts from Nardostachys root and its major ingredient Nardosinone (Nar) have sedative, adaptogen-like and anti-depressive activities [1], [2]. However, the mechanism of its action remains unknown. Li et al [3] demonstrated that Nar enhances nerve growth factor (NGF)-mediated neurite outgrowth in PC12D cells and suggested that both MAP kinase-dependent and independent signaling pathways were involved in this activity. Our previous study suggested that Nar has protective effects on primary neural cultures under the condition of oxygen-glucose deprivation *in vitro*, which is closely related to activation of extracellular signal-regulated kinase 1/2 (ERK1/2) [4]. Together, these findings show that Nar has broad effects on the nervous system, which may underlie the clinical efficacy previously demonstrated for Nardostachys root.

This study investigated the effects of Nar on neural stem cells (NSCs) isolated from mouse embryonic cerebrums. NSCs proliferation was measured using a cell counting kit-8 (CCK-8) assay, bromodeoxyuridine (BrdU) incorporation and flow cytometry; migration was observed using the neurosphere method; and differentiation was monitored with cellular specific antigens. To investigate the possible signaling pathways responsible for its effect, the ERK-cAMP related element binding protein (CREB) pathway was studied. We found that Nar has the potential to increase the proliferation of NSCs and stimulates them to selectively differentiate into neurons and oligodendrocytes. These effects may occur due to stimulation of ERK1/2 and CREB phosphorylation.

## Materials and Methods

### Animals and Chemicals

CD1 pregnant (embryonic day 14) mice were purchased from Vital River Laboratory Animal Technology Co., Ltd, Beijing, China. The certificate number was SCXK (Jing) 2011-0011. The protocol was approved according to the guidelines of the Animal Ethics committee at Beijing University of Chinese Medicine, China. All efforts were made to minimize animal suffering and to reduce the number of animals used for the experiments.

Complete Embryonic NeuroCult™ Proliferation Medium, Complete Embryonic NeuroCult™ Differentiation Medium, NeuroCult™ Chemical Dissociation Kit and heparin were from Stem Cell Technologies, CA. Recombinant human epidermal growth factor (rhEGF) and recombinant human basic fibroblast growth Factor (rhbFGF) were from Peprotech, UK. Cell Counting Kit-8 (CCK-8) was from Dojindo Molecular Technologies, Japan. Other reagents were obtained from Sigma, USA, unless otherwise specified in the text.

### Primary neurosphere culture and subculture of neural stem cells

NSCs were isolated from embryonic day 14 (E14) cerebrums of CD1 mice. Briefly, gestational day 14 mice were sacrificed, and whole brains were removed from the embryos. The cerebrums were dissected, washed with cold PBS and then transferred to a 15 mL tube containing 0.25% trypsin. After incubation at 37°C for 15 min, 15 mL of a complete proliferation medium containing Complete Embryonic NeuroCult™ Proliferation Medium, 20 ng/mL rhEGF, 10 ng/mL rhbFGF and 2 μg/mL heparin was added. The mixture was triturated approximately 10 times. Tissues were allowed to settle for 2 min, and the supernatant was filtered through a 36 μm cell strainer. The filtrate containing the primary single cells was transferred to a T-25 cm2 flask at a density of 8×104 cells/cm2. Cells were maintained in the complete proliferation medium and cultured at 37°C in a 5% CO2 humidified incubator. The formation of neurospheres was checked daily, and 50% of the medium was changed every 2-3 days.

The cells were passaged when the neurospheres reached 100–150 μm in diameter using the NeuroCult™ Chemical Dissociation Kit, following the manufacturer's instructions. The dissociated single cells were cultured in a new T-25 cm2 flask at a density of 2×104 cells/cm2.

### Analysis of the cells viability and proliferation

Single cells were dissociated from neurospheres and seeded at 1×10^4^ cells/cm^2^ with various concentrations of Nar in 96-well plates (200 μL/well). CCK-8 assay was used at the end of different experimental periods (1 d, 2 d, 3 d) as per the manufacturer's instructions. After incubation at 37°C for 4 h, the absorbance of each well at a wavelength of 450 nm was read on an enzyme-linked immunosorbent assay reader (Thermo Labsystems, Finland). For BrdU incorporation, the dissociated single cells were plated onto 24-wells plates at 1×10^4^ cells/cm^2^, and Nar (or veihcle) and BrdU (10 μM) were added to the medium at the same time. After 3 d incubation, the cells were used for BrdU detection with immunofluorescence and confocal laser imaging. For ERK1/2-CREB analysis, the cells were seeded in T-25 cm^2^ flasks at 1×10^4^ cells/cm^2^. Nar or PD98059 was added accordingly. The protein was extracted for western blot analysis after 3 d incubation.

### Analysis of the cell cycle by flow cytometry

The dissociated single cells were seeded in T-25 cm^2^ flasks at 1×10^4^ cells/cm^2^ (10 mL, 2.5×10^4^ cells/mL). After treatment with Nar for 3 d, cells were pelleted at 300 g for 5 min and fixed with 1 mL 70% cold ethanol. After ethanol fixation, cells were pelleted and washed once with PBS. Pelleted cells were stained by adding 500 μL PBS containing 50 μg/mL propidium iodide and 100 μg/mL RNAase. The cells were stained at 4°C for 30 min and detected using a Beckman Coulter FC 500. The results were analyzed by the ModFit software.

### Cells migration analysis

Following the method used by Ishido and Suzuki [5], three to five neurospheres (100–300 μm in diameter) were seeded in poly-D-lysine and laminin coated coverslips for 4 h to allow neurospheres to attach. For comparisons between groups, neurospheres with similar diameter (around 250 μm) was used for this study. The cells were then exposed to a variety of concentrations of Nar. After 24 h of incubation, the migration distance of cells was measured from the edge of the sphere, using Image-Pro Plus software. Immunostaining for nestin, microtubule-associated protein-2 (MAP2), and glial fibrillary acidic protein (GFAP) were also performed.

### Induction of cells differentiation

The neurospheres were harvested and dissociated into single cell suspensions. The cells were plated onto a poly-D-lysine and laminin coated 24-well plate containing 12 mm coverslips (IWAKIA, Japan) at a density of 5×10^4^/cm^2^ with Complete NeuroCult™ Differentiation Medium. Nar or vehicle was added to the culture medium after cells seeding. Differentiation was monitored after 7 and 14 days treatment.

### Immunostaining and confocal laser imaging

Cells were fixed in 4% paraformaldehyde (PFA) for 30 min at room temperature. After fixation, the cells were washed with PBS and permeabilized with 0.25% TritonX-100 for 25 min. Non-specific binding sites were blocked with normal goat serum (NGS) for 1 h. The dilutions of primary antibodies were as follows: mouse anti nestin 1∶500 (Abcam, UK), rabbit anti MAP2 1∶50 (Cell Signaling Technology, USA), mouse anti myelin basic protein (MBP) 1∶500 (Cell Signaling Technology, USA), rabbit anti GFAP 1∶200 (Zhongshanjinqiao, China), mouse anti BrdU 1∶200 (Zhongshanjinqiao, China). For BrdU detection, cells were permeabilized and epitopes were retrieved by 2 M HCl treatment for 30 min, followed with borax buffer (0.1 M Na_2_B_4_O_7_, pH 8.5) for 10 min. The cells were incubated with primary antibodies at 4°C overnight. Cells were then washed with PBS and probed with TRITC-labeled anti mouse secondary antibody and FITC-labeled anti rabbit secondary antibody (1∶200, Zhong Shan Jin Qiao, China). 4',6-diamidino-2-phenylindole (DAPI, Southern Biotech, USA) was used to stain nuclei in the final step. Confocal images were processed with Volocity Demo software.

### Western blot analysis

Cells were harvested in lysis buffer [50 mM Tris (pH 7.5), 150 mM NaCl, 1% NP40, 0.5% sodium deoxycholate, 1 mM EDTA and 0.1% SDS] containing a protease inhibitor cocktail. Protein concentrations were determined by BCA protein assay. Protein (50 μg) was electrophoresed on a 10% SDS-polyacrylamide gel, transferred to a polyvinylidene difluoride (PVDF) membrane (Millipore, USA) and incubated with primary antibodies at 4°C overnight. After washing, the membranes were incubated with horseradish peroxidase-conjugated secondary antibodies for 1 h. Immunoreactive bands were visualized using an enhanced chemiluminescence kit (Santa Cruz, CA). Relative densities of the bands were determined by image analysis with Image-Pro Plus software. Primary antibodies: rabbit anti ERK1/2, rabbit anti p-ERK1/2, rabbit anti CREB, rabbit anti p-CREB (1: 2000) and mouse anti GAPDH (1: 4000) (all from Cell Signaling Technology, USA).

### Statistical analysis

All data are expressed as the mean ± SEM from three experiments. Statistical analysis was performed by one-way ANOVA, followed by Student-Newman-Keuls test. The statistical significance of differences is set at P<0.05. The Western blot experiment was repeated three times. The sample size for all other quantitative study was ten with three times repetitions.

## Results

### Validation of NSCs multipotency

**Figure 1:**
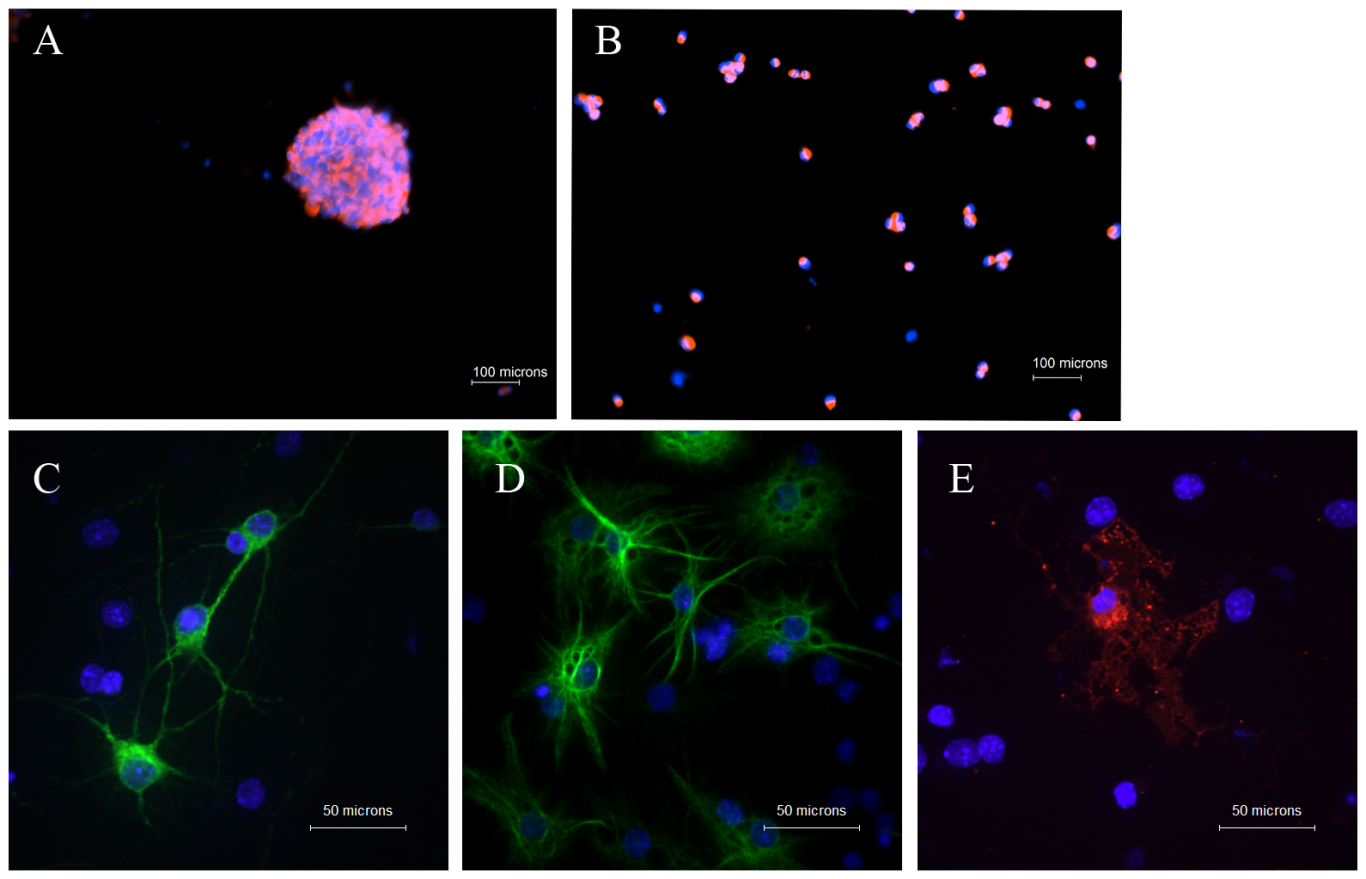
Validation of NSCs multipotency. NSCs from embryonic day 14 (E14) cerebrums of CD1 mice were cultured for 7 d and subcultured for another 7 d. The neurosphere (A) and the dissociated cells (B) from the passage 1 cultivation were stained with nestin (red fluorescent) and DAPI (blue fluorescent) and detected by immunofluorescent assay. When NSCs from passage 1 were cultured under differentiation conditions for 7 d, MAP2 positive (C, green fluorescent), GFAP positive (D, green fluorescent) and MBP positive (E, red fluorescent) cells are found. All nuclei are stained with DAPI (blue fluorescent).

In this study, cells isolated from the cerebrums of embryonic day 14 mice began to form neurospheres on the fifth day of culturing. The volume of the neurospheres, with round peripheries and bright phase contrast, was increased during the culturing process. Both the neurosphere and its dissociated single cells were nestin positive at an average rate of 90%, as show in Fig. 1A and 1B. After NSCs were cultured under differentiation conditions for 7 d, MAP2 positive, GFAP positive and MBP positive cells were detected, indicating the presence of mature neurons, astrocytes and oligodendrocytes, respectively (Fig. 1C, 1D, 1E). These results validated the multipotency of the culturing NSCs.

### Nar promotes the proliferation of NSCs

**Figure 2:**
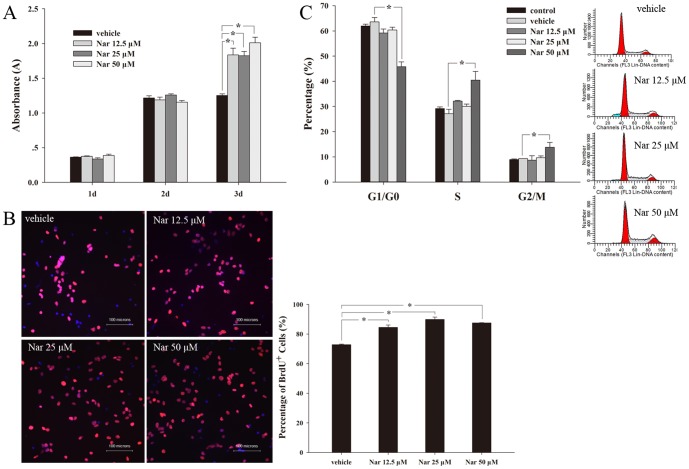
Nar promotes the proliferation of NSCs. Seeded in plates or flasks at 1×10^4^ cells/cm^2^, NSCs were incubated with varying concentrations of Nar. After culturing separately for 1 d, 2 d or 3 d, a cell counting kit-8 (CCK-8) assay was used to examine cell viability. After 3 d of treatment with Nar, cell viability was enhanced compared with vehicle (0.25% DMSO) (A). The bromodeoxyuridine (BrdU) incorporation method in combination with immunofluorescent staining (BrdU: red fluorescence, DAPI: blue fluorescence) revealed that Nar could raise the number of BrdU-positive NSCs after 3 d (B). Flow cytometry results showed that 50 μM Nar increased the proportion of cells in the DNA synthesis phase after 3 d (C). The experiment was repeated three times. * P<0.05, compared with vehicle.

In the CCK-8 assay, the absorbance steeply increased in the first 2 d after culture, indicating a possible significant proliferation of cells. Nar did not show an obvious effect on absorbance in this period. After 3 d of treatment, Nar at 12.5 μM, 25 μM and 50 μM significantly increased cell viability, as shown in Fig. 2A. We then tested the proliferation as measured by BrdU (10 μM) incorporation. We found that Nar at 12.5 μM, 25 μM and 50 μM significantly increased the number of BrdU-positive cells in comparison with vehicle treated cells (Fig. 2B). These data suggest that Nar has a facilitative effect on NSCs proliferation. To investigate the influence of Nar on cell division, flow cytometry was used to analyze cell cycle on the third day of Nar treatment. The results demonstrated that the proportions of cells in S phase and G2/M phase were significantly increased by 50 μM Nar treatment compared with vehicle and that the proportion of cells in G1/G0 phase was decreased at the same time, 45.8±1.9% and 61.9±0.7%, respectively for Nar 50 μM and vehicle (Fig. 2C). These results suggest that Nar can promote DNA synthesis during cell division.

### Nar increases NSCs migration in neurosphere assay

**Figure 3:**
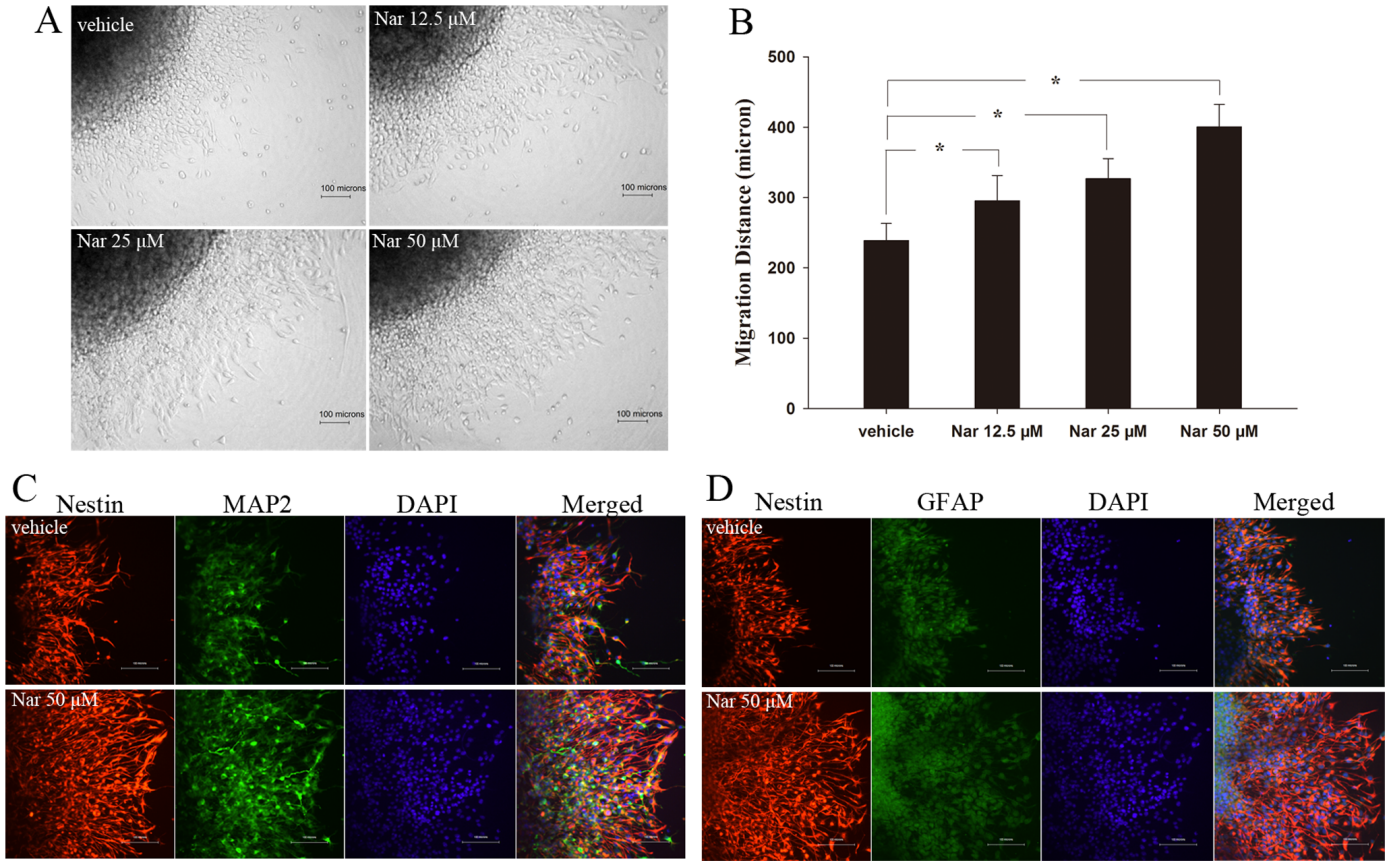
Nar increases NSCs migration in neurosphere assay. After three to five neurospheres (100–300 μm in diameter) were seeded in 24-well plates for 4 h, they were exposed to Nar. Morphological observations of cell migration were undertaken after 24 h treatment (A). The migration distance from the neurosphere was increased by Nar (B). During this period, migrating cells expressed the phenotypes of neurons and astrocytes (C, D). The experiment was repeated three times. * P<0.05, compared with vehicle.

In the first several hours of neurosphere culture, obvious cell migration from the sphere was not found. Nar was added to the medium 4 h after the culture. The cultures were continued for another 24 h, after which cell migration was analyzed. As shown in Fig. 3, the cells emerged from the neurosphere and migrated along the radial axis. Nar treatment significantly increased the distance of cell migration in a dose-dependent fashion (Fig. 3A, 3B). Immunofluorescence analysis demonstrated that both MAP2 and GFAP positive cells, most of which were also nestin positive, were observed in the cell population derived from the neurospheres, as shown in Fig. 3C, 3D. Interestingly, we found a good percentage of migrated cells stained with MAP2, but which were not nestin positive, indicating a terminal differentiation of NSCs to neurons. However, we rarely observed cells in the migrated population that were only GFAP positive, which may suggest astrocytic differentiation occurs at a later stage.

### Nar induces NSCs to selectively differentiate to neurons and oligodendrocytes

**Figure 4:**
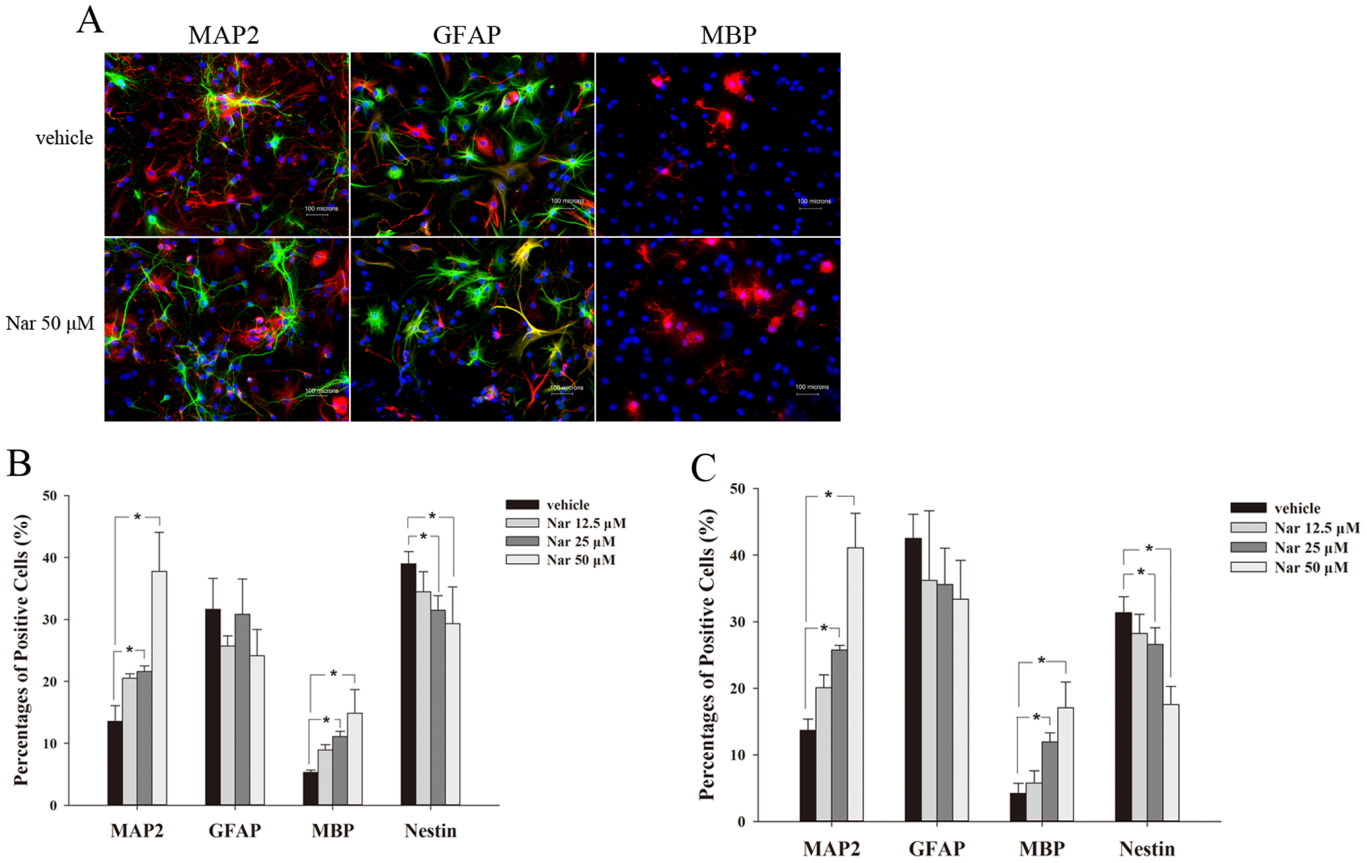
Nar selectively induces NSCs differentiation to neurons and oligodendrocytes. Incubated for 7 d, NSCs seeded in 24-well plates at 5×10^4^/cm^2^ differentiated into neurons, oligodendrocytes and astrocytes. Treatment with 25 or 50 μM Nar significantly increased the proportion of neurons and oligodendrocytes (A, B). Similar effects were observed after 14 d in differentiation culture (C). The experiment was repeated three times. * P<0.05, compared with vehicle.

To determine whether Nar could selectively induce differentiation of NSCs, we examined three types of CNS cells: neurons, oligodendrocytes and astrocytes. After 50 μM Nar treatment for 7 d, the NSCs underwent differentiation with an obvious preference for becoming neurons and oligodendrocytes. However, Nar did not show a marked influence on astrocyte differentiation (Fig. 4A, 4B). To confirm the effects of Nar on NSC differentiation, the cells were cultured for another 7 d. The results showed that in the presence of 25 μM or 50 μM Nar, the proportion of neurons (MAP2 positive) and oligodendrocytes (MBP positive) were consistently higher than in vehicle treated cultures. Interestingly, the proportions of nestin positive cells significantly decreased after 7 d treatment with 25 μM or 50 μM Nar and decreased furthered after a total of 14 d Nar treatment (Fig. 4C). These results suggest that Nar may modulate the selective differentiation of NSCs to neurons and oligodendrocytes, fostering their maturation.

### Nar regulates the ERK1/2-CREB signaling pathway in NSCs

**Figure 5:**
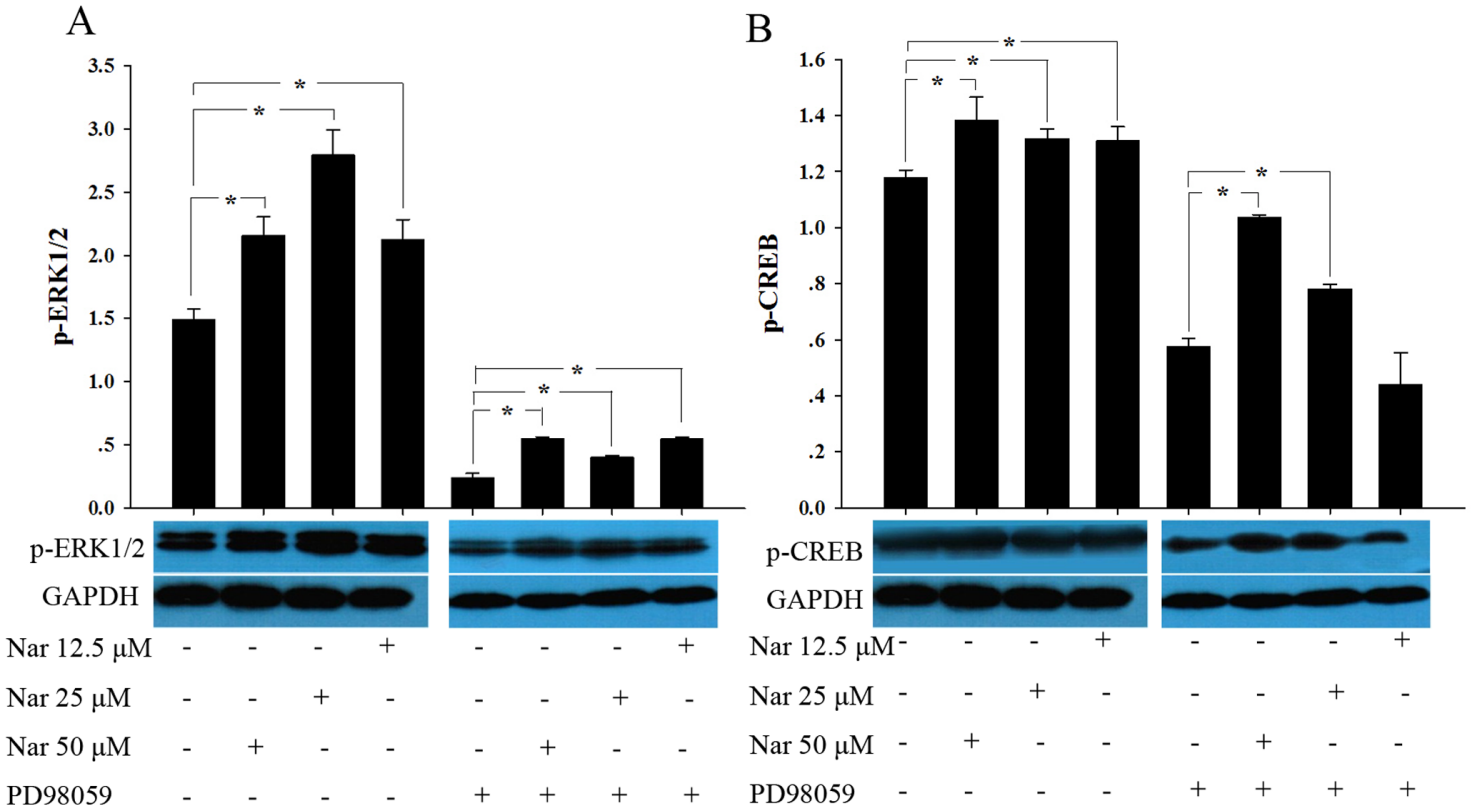
Nar regulates ERK1/2-CREB signaling in NSCs proliferation. Western blot was used to detect the expression of p-ERK1/2 and p-CREB in NSCs after treatment with Nar for 3 d. We found that Nar could promote the expression of p-ERK1/2 (A) and p-CREB (B), which was inhibited partly by PD98059, an inhibitor of mitogen-activated protein kinase kinase 1 (MEK1). The western blot experiment was repeated three times. * P<0.05, compared with vehicle.

To investigate the possible mechanisms by which Nar affects NSCs, the ERK-CREB signaling pathway was monitored using PD98059, an inhibitor of mitogen-activated protein kinase kinase 1 (MEK1). In proliferating NSCs, treatment with PD98059 (10 μM) reduced the phosphorylation of ERK 1/2, and a subsequent reduction in p-CREB was also observed. Nar itself could significantly promote the expression of p-ERK1/2 and p-CREB without influencing the expression of total ERK1/2 and CREB. Nar treatment compromised the inhibitory effect of PD98059 on ERK-CREB signaling (Fig. 5). Similar effects were also observed in the NSC differentiation study (data not shown).

## Discussion

In this study, we have found that Nar promotes the proliferation of NSCs and increases cell migration distance in a dose-dependent manner. Nar also induces the selective differentiation of NSCs to neurons and oligodendrocytes and fosters NSCs maturation.

A neurosphere is not a homogeneous collection of NSCs, instead, it is composed of several heterogeneous cell populations, such as neural stem cells, progenitor cells and dead cells [6], [7], [8]. In this study, we demonstrate that the NSCs proliferation surge occurred in the first several days after culture, at the time that most of the NSCs remained as individual cells, although neurospheres appeared sporadically. Both BrdU and cell cycle studies suggest that Nar promotes the proliferation of NSCs after 3 d of treatment.

Generally, neurosphere cells have both radial and tangential migration, depending on the molecular presence in the medium [9]. In this study, we found that most cells from neurospheres have radial migration, which was significantly facilitated by the presence of Nar in a dose-dependent manner. After 24 h of migration, a great number of projecting MAP2 cells were not stained with nestin, particularly when treated with Nar, although there were still many MAP2 and nestin double-stained cells present. The results indicate that after 24 h, some of the NSC-derived neurons were approaching maturation. Interestingly, we rarely observed cells positive for GFAP alone in the migrating population, with almost all GFAP cells also being nestin positive. We also found that migrating GFAP cells did not possess astrocytic projection, which suggests that migrating GFAP cells are still at either the stem cell or progenitor cell stage. The results also indicate that the differentiation of NSCs to astrocytes occurs at a later stage than neuronal differentiation, as previously reported by others [10], [11].

The stem cells and progenitor cells in neurospheres could give rise to neurons, astrocytes and oligodendrocytes when dissociated and cultured in an appropriate medium. It is possible that the phenotype of some cells has already been decided prior to dissociation [7]. In this study, we found that after 7 d culturing in differentiation medium, most of the differentiated cells lost their reactivity to the nestin antibody, which suggests the cells are approaching maturation, as we indicated in the migration study. However, we still found a good percentage of cells, either MAP positive or GFAP positive, that react to the nestin antibody. This finding is supported by Temple [10], who found that the neurogenic phase usually takes 5 d, and the gliogenic phase takes 10 d. Nar could also increase the percentage of MAP2 and MBP cells after 7 d and 14 d treatment, with no obvious influence on GFAP cells. This is also the case after 14 d of treatment. Interestingly, nestin positive cells was significantly decreased after 14 d of Nar treatment compared with vehicle. The results suggest that Nar selectively enhances differentiation of NSCs to neurons and oligodendrocytes, with facilitating the maturation of NSCs under these differentiation conditions.

ERK-CREB signaling plays a very important role in stem cell survival [12]–[15]. Modulating CREB activity during early development can cause significant defects in neural proliferation [16]–[18]. In this study we found that Nar could significantly increase the expression of p-ERK1/2 and subsequently p-CREB without influencing the total expression of ERK and CREB, a finding that is also supported by our previous work on primary neural cultures [4]. When MEK1 phosphorylation was inhibited by PD98059, both p-ERK and p-CREB level decreased as expected. Nar significantly attenuated the inhibitory effect of PD98059. Together, these results indicate that Nar may influence NSCs through ERK-CREB signaling. The exact mechanisms by which this occurs require further study.

In conclusion, we have identified a novel Nar-mediated regulation of NSC proliferation, migration and selective differentiation. However, owing to the complex constitution of the neurosphere and its associated limitations, clarifying the mechanisms of Nar action on cellular activities requires more work. By using fluorescence-activated cell sorting (FACS) with specific markers and measuring the protein and gene expression level changes in signaling pathways within specific subsets of NSCs, we will better understand the mechanism by which Nar affects NSC activities.
